# The effects of the COVID-19 pandemic on acute and elective peripheral nerve surgery

**DOI:** 10.1007/s00508-026-02757-1

**Published:** 2026-05-08

**Authors:** Eva Placheta-Györi, Oliver Bernhart, Elias Ludescher, Christine Radtke

**Affiliations:** Department of Plastic, Reconstructive and Aesthetic Surgery, Medical University of Vienna, Spitalgasse 23, 1090 Vienna, Austria

**Keywords:** Neuropathy, Neurorrhaphy, Autograft, Reconstruction

## Abstract

**Objectives:**

Nerve injuries and regeneration after peripheral nerve surgery are time-sensitive, therefore longer waiting times and delayed treatment could impact patient management and functional results. This study aimed to investigate the effects of the COVID-19 pandemic on acute and elective peripheral nerve surgery during the strict first lockdown measures.

**Material and methods:**

In this retrospective study 352 patients who underwent peripheral nerve surgery in a university-based setting in an academic center during the COVID-19 lockdown (March 2020 to March 2021) were included. The pre-COVID-19 period of March 2018 to March 2019 served as a baseline for peripheral nerve procedures at the same department. Patient demographics, type of peripheral nerve lesion, affected peripheral nerves, operative treatment, postoperative complications and length of hospital stay were assessed. Acute indications and elective peripheral nerve surgery were assessed in 2 separate groups to evaluate influences of COVID-19 restrictions during the first lock-down period.

**Results:**

A total of 198 patients underwent peripheral nerve surgery during the pre-COVID-19 period compared to 154 patients during the COVID-19 period. The most common forms of surgery were peripheral nerve decompression procedures, which were significantly reduced during the COVID-19 period. While an overall reduction of peripheral nerve procedures was found, the proportion of posttraumatic reconstruction and nerve tumor surgery increased during the COVID-19 period.

**Conclusion:**

The measures taken during the COVID-19 period altered the number of elective peripheral nerve surgeries. While the overall number of patients treated was reduced, urgent cases were prioritized.

## Introduction

The outbreak of the novel coronavirus SARS-CoV‑2 in late 2019 rapidly escalated into a global health crisis. The COVID-19 pandemic was declared by the World Health Organization in March 2020, after a global outbreak of a novel coronavirus SARS-CoV‑2 [1]. Strict social isolation measurements and lock-down recommendations at the beginning of the pandemic lead to the cancellation or delay of elective forms of surgery [2, 3]. These restrictions, while necessary to mitigate viral transmission and preserve intensive care resources, had far-reaching consequences for the delivery of surgical care. It has been reported that in the first 12 weeks of the COVID-19 pandemic an estimated 28.4 million surgeries were delayed or cancelled worldwide, which correlated to approximately 72% of all surgical procedures [4].

While elective surgeries were most directly affected by these measures, acute surgical care was also significantly impacted. The COVID-19 pandemic has also impacted acute surgical cases as it was found that injury patterns changed during the early periods of the pandemic. Previous studies have reported that although the overall number of surgical emergencies declined by approximately 30–35% during the first lockdown, there was a significant increase in suicide attempts and domestic accidents [5–7].

Peripheral nerve surgery was broadly impacted by the global pandemic [8] and was affected by the measures associated with the COVID-19 pandemic in both the acute and elective settings. Recommendations for triage of plastic surgery patients included peripheral nerve procedures ranging from low acuity (e.g. carpal tunnel release) to high acuity (e.g. acute nerve injuries, complex trauma, compartment syndrome) [9].

The effects of delays of surgical procedures for treating peripheral nerve conditions are of particular concern, as peripheral nerve regeneration is time sensitive. After a peripheral nerve injury, the success of surgical repair is significantly influenced by several biological challenges [10]. Among the most critical are neuronal cell death, progressive atrophy of the target muscles and the effects of chronic denervation and chronic axotomy [11–15]. These adverse processes can compromise functional recovery even when surgical reconstruction is promptly and technically well performed. Their impact is especially pronounced in injuries requiring regeneration across long distances, which prolongs the interval to target reinnervation. Prolonged regeneration intervals are known to exacerbate the degeneration of both the distal nerve stump and the denervated end organ, further reducing the likelihood of satisfactory functional outcomes [14].

Chronic denervation and axotomy have been described as independent risk factors leading to incomplete recovery following peripheral nerve surgery [14, 15]. Chronic denervation and chronic axotomy can significantly impair successful functional recovery following peripheral nerve injury depending on the timing of surgical repair and the regeneration distance [16–18]. These mechanisms have been well-characterized in preclinical studies, showing that even with technically successful repair, delays in intervention lead to markedly reduced motor unit recovery and muscle force [14, 15]. For example, a delay of 6 months can result in a 90% reduction in regenerating motor units, primarily due to a loss of motoneurons capable of axonal regrowth rather than end organ receptivity. Although some muscle fibers remain viable, this reduction exceeds the compensatory capacity of collateral sprouting [19].

Clinically, this translates to poorer outcomes in patients who undergo delayed nerve repair, particularly in injuries involving long regeneration distances, such as brachial plexus lesions [20]. These findings highlight the time-sensitive nature of nerve repair and underscore the importance of early surgical intervention to optimize motor recovery and long-term functional outcomes in clinical practice.

Even nonacute cases, such as chronic compression neuropathy are relatively time sensitive, as chronic nerve lesions lead to incomplete recovery even after successful surgical interventions [21, 22].

The aim of this study was the analysis of the effects of the COVID-19 pandemic during the early lock-down measures on acute and elective peripheral nerve surgery and to compare this time period to a representative time period before the COVID-19 pandemic.

## Material and methods

Patients treated with acute or elective peripheral nerve surgery at our university-based hospital were included in this retrospective study if the procedures were performed at the Department of Plastic, Reconstructive and Esthetic Surgery between March 2018 and March 2019 (“pre-COVID-19 group”) or between March 2020 and March 2021 (“COVID-19 group”).

Ethical approval for this study (Reference No. 1217/2022) was provided by the institutional Ethics Committee on 9 May 2022.

Surgical reports and medical histories were retrieved using the electronic patient history. The parameters assessed included patient demographics, type nerve lesion or injury and the affected nerve. The type of peripheral nerve surgery (e.g. nerve decompression, neurorrhaphy, nerve transfer, nerve autograft, allograft), length of hospital stay and follow-up time were described. Postoperative complications (minor complication healing under conservative treatment; major complications requiring surgical revision) were analyzed.

### Statistical analysis

Descriptive statistics including mean value, standard deviation and range were reported for all patient data. Parametric and non-parametric tests were used for group comparisons depending on the normal distribution of the data. The patient collective was grouped and analyzed based on the acuity of peripheral nerve lesion (acute/elective) and time period treated (pre-COVID-19 or COVID-19). A two-sided alpha of 5% was applied in all statistical tests. Programs used for the statistical analysis and graphs included SPSS Statistics (Version 25, IBM Corp, Armonk, NY, USA), Excel (Version 2310, Microsoft 365, Microsoft Corporation, 2023, Redmond, WA, USA) and GraphPad Prism 9 (Version 9.2.0, GraphPad Software, Boston, MA, USA).

## Results

### Patient demographics

**Fig. 1:**
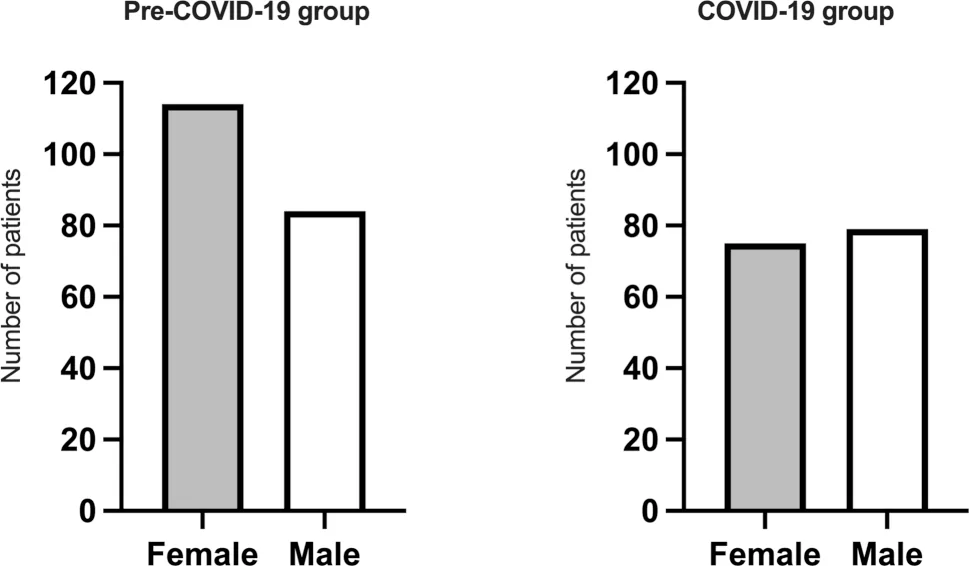
Sex distribution of patients undergoing peripheral nerve surgery in the pre-COVID-19 and COVID-19 cohorts.The figure compares the sex distribution between patients treated before the onset of the COVID-19 pandemic (pre-COVID-19 group) and those treated during the pandemic period (COVID-19 group). In the pre-COVID-19 cohort, 114 patients were female and 84 were male. In the COVID-19 cohort, 75 patients were female and 79 were male

A total of 198 patients underwent peripheral nerve surgery during the pre-COVID-19 period compared to 154 patients during the COVID-19 period. In the pre-COVID-19 group, 114 female (57.6%) and 84 male patients (42.6%) were included (Fig. 1). The mean age was 51.2 years (SD 19.1 years, range 0.9–87.2 years). In the COVID-19 group, the mean age was 48.9 years (SD 20.3 years, range 0.4–88.6 years), including 75 female (48%) and 79 male patients (51.2%). In the pre-COVID-19 group, there was a significant sex difference as more female patients were treated (*p* = 0.020) but no sex difference was observed in the COVID-19 group.

### Types of peripheral nerve lesion

Overall, the most common of peripheral nerve lesion leading to acute surgical treatment were lacerations (47.37%) as shown in Table 1. In the pre-COVID-19 group, burn-related compression injuries of peripheral nerves had the highest incidence (57.14%), while there were no burn-related cases during the COVID-19 period.

**Table 1:** Types of peripheral nerve lesions in acute surgery. The types of peripheral nerve lesions that resulted in acute surgical treatment in the pre-COVID-19 group (*n* = 14) and the COVID-19 group (*n* = 5) are shown

Lesion type	Pre-COVID-19	COVID-19	Total
Laceration	5 (35.7%)	4 (80.00%)	9 (47.37%)
Burn-related injury	8 (57.14%)	0 (0.00%)	8 (42.11%)
Acute compression	1 (7.14%)	0 (0.00%)	1 (5.26%)
Explosion injury	0 (0.00%)	1 (20.00%)	1 (5.26%)
*Total*	*14 (100%)*	*5 (100%)*	*19 (100%)*

In the elective cases, the most common type of nerve lesion was compression neuropathy (Table 2) in both the pre-COVID-19 and the COVID-19 groups; however, there was a significant reduction in the nerve compression cases in the COVID-19 group (*p* < 0.001).

**Table 2:** Types of peripheral nerve lesions in elective surgery. The types of peripheral nerve lesions that resulted in elective surgical treatment in the pre-COVID-19 group (*n* = 189) and the COVID-19 group (*n* = 148) are shown

Type of nerve lesion	Pre-COVID-19	COVID-19	Total
Nerve compression	146 (77.25%)	89 (59.73%)	235 (69.53%)
Resection of a peripheral nerve tumor	21 (11.11%)	33 (22.15%)	54 (15.98%)
Traumatic lesion of peripheral nerves	15 (7.94%)	12 (8.05%)	27 (7.99%)
Traumatic lesion of the brachial plexus	7 (3.70%)	10 (6.71%)	17 (5.62%)
Cerebral hemiparesis	0 (0.00%)	3 (2.01%)	3 (0.89%)
*Total*	*189 (55.92%)*	*148 (44.08%)*	*338 (100.00%)*

The difference in the number of surgical excisions of peripheral nerve tumors between the pre-COVID-19 and COVID-19 groups was not statistically significant (*p* = 0.134). Furthermore, no significant differences were observed in the number of procedures performed for the elective surgical treatment of traumatic peripheral nerve lesions (*p* = 0.701) or elective traumatic brachial plexus injuries (*p* = 0.359) between the two groups.

### Affected peripheral nerves

The median nerve was the most commonly affected peripheral nerve in both the pre-COVID-19 and the COVID-19 groups, followed by the ulnar nerve (Table 3). There was a significant reduction in elective median nerve surgery during the COVID-19 pandemic. A reduction of acute median nerve surgery was also found. Elective procedures involving the ulnar nerve were not significantly reduced during the COVID-19 pandemic. There was an increase in elective brachial plexus cases in the COVID-19 group, although it did not reach statistical significance.

**Table 3:** Most common affected peripheral nerves. The most commonly affected peripheral nerves treated by elective or acute surgery are shown for the pre-COVID-19 and the COVID-19 group

Affected nerve	Pre-COVID-19	COVID-19	*p*-value
**Acute PNS surgery**			
Median nerve	10	2	*0.039*
Ulnar nerve	3	0	n/a
Digital nerves II	2	1	n/a
Digital nerves III	2	0	n/a
Digital nerves IV	2	1	n/a
***Total***	***19***	***4***	***–***
**Elective PNS surgery**			
Median nerve	109	59	*<* *0.001*
Ulnar nerve	29	27	0.894
Brachial plexus	10	21	0.071
Tibial nerve	3	6	n/a
Common peroneal nerve	5	3	n/a
***Total***	***199***	***148***	–

*n/a* statistical group comparison not applicable due to group sizes *n* < 6, *PNS* peripheral nerve surgery

### Types of peripheral nerve surgery

In the pre-COVID-19 group epineural sutures of peripheral nerves were the most common surgical procedure, followed by acute median nerve decompression (Table 4). In the COVID-19 group, intrafascicular sutures and acute decompression of the median nerve were performed in 3 patients.

**Table 4:** Acute surgical procedures. Please note that the number of surgical procedures surpasses the total number of patients as some patients had more procedures performed during the surgery

Surgical procedure	Pre-COVID-19	COVID-19	Total
Decompression of the median nerve	5 (33.3%)	3 (42.8%)	8 (36.36%)
Epineural suture	8 (55.33%)	0	8 (36.36%)
Intrafascicular suture	0	3 (42.8%)	3 (13.64%)
Decompression of other nerves	2 (13.33%)	0	2 (9.1%)
Autologous nerve graft	0	1 (14.29%)	1 (4.5%)
*Total*	*15 (68.18%)*	*7 (31.82%)*	*22 (100.0%)*

The most common type of elective peripheral nerve surgery in the pre-COVID-19 group was surgical decompression of the median nerve (44.61%), followed by surgical decompression of other nerves (20.59%) as shown in Table 5. In the COVID-19 group surgical decompression of the median nerve (21.54%) and other peripheral nerves (24.10%) were the most common elective procedures.

**Table 5:** Elective surgical procedures. Please note that the number of surgical procedures surpasses the total number of patients as some patients had more procedures performed during the surgery

Surgical procedure	Pre-COVID-19	COVID-19	Total
Decompression of the median nerve	91 (44.61%)	42 (21.54%)	133 (33.33%)
Decompression of other nerves	42 (20.59%)	47 (24.10%)	89 (22.31%)
Tumor excision from peripheral nerves	25 (12.25%)	31 (15.90%)	56 (14.04%)
Autologous nerve graft	13 (6.37%)	25 (12.82%)	38 (9.52%)
Decompression of the lower extremity	18 (8.82%)	11 (5.64%)	29 (7.27%)
Reconstruction of a peripheral nerve plexus	7 (3.43%)	19 (9.74%)	26 (6.52%)
Intrafascicular suture	2 (0.98%)	4 (2.05%)	6 (1.50%)
Simple suture	6 (2.94%)	15 (7.69%)	21 (5.26%)
*Total*	*204 (51.13%)*	*195 (48.87%)*	*399 (100.0%)*

### Time to surgery

**Fig. 2:**
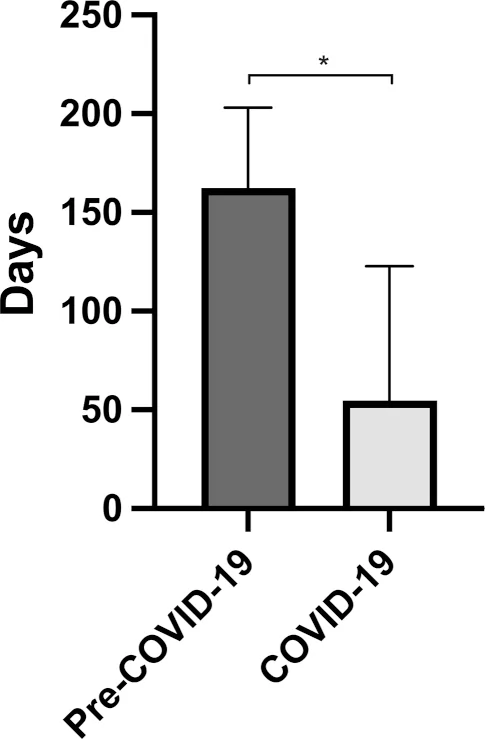
Time from initial diagnosis to operation. The mean time from the initial diagnosis of the peripheral nerve lesion to the operation significantly decreased during the COVID-19 pandemic

In elective cases the group of patients with nerve compression, the time interval between the date of initial diagnosis (at our center or before referral) and the date of surgery were significantly shorter in the COVID group at 54.7 days (SD: 68.05 days; SEM 40.7 days) compared to the pre-COVID group at 162 days (SD: 528.1 days; SEM 5.7 days) (*p* = 0.0096, Fig. 2).

The interval between the date of surgery and the date of discharge was significantly longer in the COVID-19 group at 4.5 days (SD: 23.2 days, range: 0–272 days) compared to 2.2 days (SD: 5.5; range 0–69 days) in the pre-COVID-19 group (*p* = 0.034).

### Postoperative complications

There were no postoperative complications in acute peripheral nerve surgery in either group. A total of 9 patients who underwent elective peripheral nerve surgery experienced postoperative complications within the first 12 weeks following surgery. Of these 9 complications 6 occurred in the pre-COVID group (2.9% of all procedures) and 3 in the COVID group (1.5% of all procedures). In the pre-COVID group 3 complications were classified as “severe” and 3 as “mild.” All three complications in the COVID group were categorized as “mild.” There was no statistically significant difference in the number of complications between the two groups (*p* = 0.517).

## Discussion

The aim of this study was to assess how the COVID-19 pandemic influenced elective and acute peripheral nerve surgery at the Department of Plastic, Reconstructive and Esthetic Surgery of a university-based hospital. Surgical volume, case profiles, patient demographics, complication rates, and perioperative timelines were analyzed for two time periods: pre-COVID-19 (March 2018 to March 2019) and during the COVID-19 pandemic (March 2020 to March 2021).

The global surgical landscape changed dramatically in response to the COVID-19 pandemic. Reduced operating room availability, staff redeployment, and infection control protocols led to the triaging of surgery based on urgency [2]. Studies have shown that regions and specialties with high proportions of elective procedures are especially affected by delays or cancellations of surgery [3, 4]. Despite their elective classification many of these procedures, although not urgent, are not optional. Delays can negatively impact functional outcomes, especially in peripheral nerve surgery [5, 23].

Nerve repair procedures, although often scheduled in a non-emergency context, must frequently be performed within a critical window to prevent permanent functional loss. As MacKay et al. highlighted, postponing such procedures during the COVID-19 pandemic risked exceeding the optimal timeframe for reinnervation, thereby worsening postoperative outcomes [8].

In this study, the total number of elective peripheral nerve surgeries decreased significantly by over 20% in the COVID-19 cohort compared to the pre-COVID-19 group. The decrease of peripheral nerve procedures in the early phases of the COVID-19 pandemic was also described by other specialized centers [24].

Similar trends were observed internationally, with reported reductions in plastic surgery volumes of up to 87.6% during early lockdown phases [25]. The reduced volume is likely due to prioritization of urgent cases, reallocation of resources to COVID-19 care [26] and patients’ fear of hospital-acquired infections [27]. This elective surgical deferral also contributed to a backlog that strained healthcare systems post-pandemic [28].

In contrast to international reports where the volume of acute surgical cases remained relatively stable [29], our study found a marked decrease in acute peripheral nerve interventions, from 14 in the pre-COVID-19 group to 5 during the pandemic, although the case numbers are very low in the current series. Possible referrals of patients with acute nerve injuries to nonacademic local hospitals could not be analyzed in this retrospective study but may account for the reduction of acute peripheral nerve injuries treated at our center during the COVID-19 period. This should be addressed in future multicenter studies across all medical specialties treating patients with peripheral nerve injuries.

Previous studies have shown a significant shift of injury patterns during the COVID-19 pandemic [6]. The data of the current study suggest that burn injuries were reduced as work-related injuries were less common due to the lockdown measures. Larger multicenter studies would, however, be required to explore this hypothesis.

A German study found that hospital admissions of acute trauma cases increased during the pandemic [30] and a study from a university-based center in Austria also reported an increase in acute interventions during lockdowns [31]. This decline at our center may reflect localized trends, including reduced trauma volume due to lockdowns and fewer occupational injuries. Our findings are consistent with multiple international reports describing a decrease in occupational and traffic-related injuries and an increase in domestic and fall-related incidents during lockdown periods [7]. A change of injury patterns during the early periods of the COVID-19 pandemic was consistently described in international studies [32]. A large meta-analysis found that although overall major trauma cases declined by 24%, there was a relative increase in trauma from gun violence and suicide attempts, with unchanged trauma severity and mortality [33]. This is consistent with international findings reporting increased domestic and fewer occupational or motor vehicle-related injuries [6, 30].

In the current study, the time between the initial diagnosis and the surgery was significantly reduced during the COVID-19 period. This may be due to the changes in the operating lists overall as peripheral nerve cases were prioritized due to the time-sensitive nature of nerve regeneration [8].

Shorter hospital stays were strongly encouraged during the pandemic to reduce infection risk and related mortality [23]. While other studies reported reduced length of stay for elective procedures [34, 35], our data surprisingly showed longer inpatient stays in the COVID cohort. This discrepancy may be explained by a change in the case mix of procedures: during the pandemic, significantly fewer carpal tunnel syndrome releases (typically performed as same-day surgery) were carried out, whereas the proportion of more complex peripheral nerve procedures requiring longer postoperative recovery increased, thereby resulting in prolonged inpatient stays in the COVID cohort.

Despite reductions in surgical volume and changes to clinical workflows, no significant increase in postoperative complications was observed in either the elective or acute surgery cohorts during the pandemic. These findings are consistent with reports from other European centers [31].

Data from Sweden also suggested that older patients were disproportionately affected by surgical delays [36]. This study analyzing neurosurgical procedures during the COVID-19 pandemic found a significant reduction of peripheral nerve procedures in older patients [36]. Our findings may not fully capture this demographic impact due to the limited sample size but highlight the need to evaluate access disparities in future analyses.

Several study limitations must be acknowledged. This was a retrospective, single-center study with a limited sample size, especially in the acute nerve injury cohort. The diversity of included diagnoses and procedures created multiple small subgroups, which reduced the statistical power. Additionally, retrospective data may be incomplete and potential confounders could not be fully controlled. Finally, the study only included patients who underwent surgery and cannot account for those where procedures were delayed or cancelled. Future studies should address the functional outcomes and the effects of possibly delayed treatment of patients with peripheral nerve lesions. National and international multicenter studies are required to further analyze changes in referral patterns. The long-term effects of measures taken during the COVID-19 period should be assessed for patients with peripheral nerve lesions to prepare peripheral nerve surgeons for future crisis events.

## Conclusion

This study provides a comprehensive analysis of the impact of the COVID-19 pandemic on peripheral nerve surgery at a university-based tertiary referral center. Both elective and acute peripheral nerve surgeries declined during the early lockdown measures of the COVID-19 pandemic. Notably, the pandemic led to shifts in injury mechanisms and patient demographics. The study underscores the need for adaptable clinical pathways that ensure timely access to surgical care, even under restrictive conditions. Future research with larger multicenter datasets is needed to further assess long-term functional outcomes and guide preparedness strategies for future health emergencies.

## Data Availability

The underlying research data can be accessed by contacting the corresponding author at eva.placheta@meduniwien.ac.at

## Abbreviations

SD: Standard deviation
SEM: Standard error of mean
SARS: Severe acute respiratory syndrome
COVID-19: Coronavirus disease 2019
